# Ten Essential Practices for Developing or Reforming a Biostatistics Core for a NCI Designated Cancer Center

**DOI:** 10.1093/jncics/pky010

**Published:** 2018-04-28

**Authors:** Madhu Mazumdar, Erin L Moshier, Umut Özbek, Ramon Parsons

**Affiliations:** 1 Institute for Healthcare Delivery Science, Mount Sinai Health System, New York, NY; 2 Center for Biostatistics, Department of Population Health Science and Policy, Icahn School of Medicine at Mount Sinai, New York, NY; 3 Department of Oncological Sciences, Icahn School of Medicine at Mount Sinai, New York, NY; 4 Medicine, Hematology and Medical Oncology, Icahn School of Medicine at Mount Sinai, New York, NY; 5 Biostatistics Shared Resource Facility, Icahn School of Medicine at Mount Sinai, New York, NY; 6 Tisch Cancer Institute, Icahn School of Medicine at Mount Sinai, New York, NY

## Abstract

There are 69 National Cancer Institute (NCI) designated Cancer Centers (CCs) in the United States. Biostatistical collaboration is pivotal in cancer research, and support for a cancer biostatistics shared resource facility (C-BSRF) is included in the award. Although the services and staff needed in a C-BSRF have been outlined in general terms and best practices for biostatistical consultations and collaboration in an academic health center have been agreed upon, implementing these practices in the demanding setting of cancer centers interested in pursuing or maintaining NCI designation remains challenging. We surveyed all C-BSRF websites to assess their organizational charts, governance, size, services provided, and financial models and have identified 10 essential practices for the development of a successful C-BSRF. Here, we share our success with, and barriers to, implementation of these practices. Showcasing development plans for these essential practices resulted in an NCI score of “Excellent to Outstanding” for our C-BSRF in 2015, and performance metrics in 2016–2017 demonstrated notable improvement since our original Cancer Center Support Grant (CCSG) application in 2014. We believe that the essential practices described here can be adapted and adjusted, as needed, for CCs of various sizes and with different types of cancer research programs.

There are 69 National Cancer Institute (NCI) designated Cancer Centers (CCs) in the United States. Biostatistical collaboration is pivotal in cancer research, and a biostatistics shared resource has specifically been requested in the grant guidelines since the launch of the NCI Cancer Center Program in 1971. (https://grants.nih.gov/grants/guide/pa-files/PAR-17-095.html).

The multidisciplinary nature of cancer research frequently raises novel design and analytic challenges, and biostatisticians play a key role in addressing these challenges. Examples include use of Bayesian analysis for a phase II clinical trial with simple and complex end points, design of a two-stage confirmatory trial of personalized medicines to estimate treatment effect, a comparison of statistical methods for the study of etiologic heterogeneity, and mixture models for undiagnosed prevalent disease and interval-censored incident disease, as applied to cohorts derived from electronic health records (1–4).

NCI Cancer Center designation can be extremely important for academic health centers (AHCs). Approximately 75% of successful investigator-initiated grants awarded by the NCI are awarded to NCI designated Cancer Center investigators (https://www.cancer.gov/research/nci-role/cancer-centers). Biostatistics collaborators contribute in large measure to these results; the number of biostatistics faculty is shown to have a statistically significant positive association with the amount of National Institutes of Health (NIH) awards (5).

While biostatistics is central to the mission of cancer centers, and participation by biostatisticians in collaborative activities is eligible for NCI support, each center has the flexibility to propose the specific functions it wishes to have funded. Applicants for a new proposal or renewal are asked to describe major services, cost-effectiveness, management structure, operational policies, prioritization of use, and staff qualification. But the guidelines are not specific (6). It can be difficult to figure out what kind of center-specific biostatistics resource to build given this limited guidance. AHCs must look to the experience and results of other cancer centers to decide what type of facility will be both appropriate for their institution and competitive for NCI Cancer Center designation.

While most AHCs have developed statistical units that collaborate with researchers in all fields across the institution, the existence of such a unit does not guarantee a strong C-BSRF. Although best practices for biostatistical collaboration in an academic health center have been agreed upon, implementing these practices in the demanding setting of cancer centers in pursuit or maintenance of NCI designation requires special efforts and strategies (7).

A few papers offering guidelines on biostatistics unit development are available and useful, but specific strategies for developing a C-BSRF are uncommon, and none are comprehensive (6,8–10). No centralized survey of C-BSRFs currently exists. Our Core team at the Tisch Cancer Institute (TCI) at the Icahn School of Medicine at Mount Sinai (ISMMS) surveyed the websites of all NCI designated C-BSRFs to assess their organizational structures, size, financial models, and primary functions/services to identify essential practices for building a successful C-BSRF (Table 1). Our C-BSRF was integrated into the TCI at the institute’s inception in 2007 but was successfully reformed with the essential features outlined in this paper in 2014. We received a rating of “excellent to outstanding” in 2015 when the TCI became an NCI designated CC. Implementation of these essential elements has improved collaboration, productivity, and grantsmanship.

**Table 1.** Primary services/functions provided by Biostatistics Shared Facilities for Cancer Centers

Services provided for collaboration supported by Cancer Center Support Grants (CCSGs) Investigator-initiated trials protocol development and review Grant development and review Teaching short courses in experimental design and analysis methodology Mentoring for K award or Young Investigator awards
Services provided for collaboration supported by grants and contracts Data analysis Assistance with manuscript writing and review Assistance with research conferences (eg, data analysis and preconference critiques of fellows’ presentations) Assistance with identification of research gaps in order to initiate research Assistance with identifying funded grants and program announcements for grant submission planning Assistance with journal clubs and paper review (from a methodology perspective)

## 1. Director’s Full Involvement in Cancer Center Leadership

To ensure that the C-BSRF will be fully integrated into, and able to adequately support, the research efforts of the center investigators (CIs), the Core’s Scientific Director should be a member of the center’s leadership team. The Director should promote and facilitate early involvement of C-BSRF members on cancer research teams to enable significant impact by each member on research methodology practices in their group, training in their collaborators’ fields of research, the statistical methodologies to be employed, and productivity of their group regarding publication and grantsmanship. New and small C-BSRFs typically have one Director, while larger Cores may have both a Scientific Director and a Technical or Managing Director. Our C-BSRF was led in its first three years by a single individual who served as both Scientific and Technical Director. Subsequently, a senior biostatistician was promoted to the position of Managing Director with responsibility of overseeing the protocol review system.

The C-BSRF Director’s full participation in the administration of the center sends the message to members that full participation by the C-BSRF is the desired goal. Our C-BSRF Director meets quarterly with the TCI Director to discuss operational issues to ensure that internal systems and processes are effective and meets monthly with the entire institute leadership team, which comprises key members of existing and newly developing programs, administrators, and Directors of other shared facilities. Meetings are devoted to strategizing about the execution of plans in the current NCI CC grant and plans for submission of renewal of the grant. Involvement in these discussions allows the C-BSRF Director to provide input on plans for the center’s growth and to identify committees on which C-BSRF members should be represented and processes that may need adjustment to incorporate the input of biostatisticians.

The C-BSRF Director meets weekly with the Core’s staff to discuss the center’s progress and plans to help Core members become team scientists who understand the inner workings of the CC and feel responsible for enhancing collaborative productivity.

## 2. Biostatisticians Fully Dedicated to Cancer Research and Supported by a Team

We based our P30 proposal on the size (ie, staffing) and distinguishing practices of the highest-performing cancer centers. Those ranked in the top 10 by *US News and World Report* (https://health.usnews.com/best-hospitals/rankings/cancer) have larger C-BSRFs, consisting on average of 15 PhD biostatisticians (interquartile range [IQR] = 12–17) and 13 MS biostatisticians (IQR = 10–14). Our TCI-C-BSRF began with 4.30 full-time equivalent (FTE) biostatisticians, with 0.90 FTEs supported through the Cancer Center Support Grant (CCSG). Although the %FTE request for a C-BSRF on a CCSG application will depend specifically on the types of CCs, we think five FTEs is a good start. Our Core has now expanded to 6.9 FTEs, with 1.6 FTEs supported through the CCSG. Our C-BSRF also hired a part-time (30%) administrative assistant to assist with website management, appointment scheduling, arrangements for educational/training activities, and electronic tracking. In retrospect, we believe that 0.5, rather than 0.3, would have better covered our administrative needs, and we recommend that sufficient resources be devoted to ensure effective management of the website, productivity reporting, and scheduling.

CCs associated with an academic health center have biostatisticians working in a variety of fields. We believe that the majority of C-BSRF biostatisticians should be fully dedicated (100%) to cancer research. It is essential to dedicate deep effort toward understanding the clinical questions posed by investigators in the center and full commitment to designing studies appropriate to the specific biologic and/or disease focus (eg, immunology, gene expressions and regulation, epidemiology, developmental therapeutics, breast cancer, leukemia, lung cancer, melanoma, prevention and control, etc.). It is also important to ensure the availability of an adequate number of biostatisticians to enable each biostatistician to develop expertise in a few specific types of cancer and/or the technologies related to them (eg, microarray data and flow cytometry data for melanoma and prostate cancer). Scientific support for broader quantitative science areas (eg, health economists, decision scientists, and survey researchers) may be useful, depending on the research interests of the investigators in the center. CCs should consider CIs’ research needs in determining the quantitative expertise that best supports current and planned work.

While some CCs have a Bioinformatics Core integrated into the C-BSRF, the majority of NCI designated CCs have separate Bioinformatics Cores. We are currently in the process of developing such a Core. Best practices for a CC Bioinformatics Core are consistent with many of the essential practices for a C-BSRF: scientific collaborations, careful selection of the most relevant data sets and methods for a given research problem, and regular communication among all stakeholders. Given the increasing need for the analysis of large data sets from public resources and the development and testing of algorithms related to specific cancer research studies, we recommend the development of a separate Bioinformatics Core (11,12).

## 3. Programmatic Collaborations

TCI comprises four cancer research programs: cancer mechanisms, immunology, liver cancer, and prevention and control. Each program is paired with a junior biostatistician and at least one senior (PhD) biostatistician. Senior biostatisticians engage in specific program collaborations and attend works-in-progress meetings with investigators in their program areas. Junior biostatisticians frequently take the lead in the interim analysis of the data and perform the final analysis for manuscript submission. At the outset of collaboration, both groups spend significant time learning about each other’s areas of expertise. This investment results in more productive research over time. TCI C-BSRF members attend monthly Disease-Focused Groups (DFG) and final Protocol Review Monitoring Committee (PRMC) meetings to make recommendations on study design.

We recommend that members of the BSRF sit on the committees responsible for review of protocols with the goal of ensuring that statistical designs and analysis plans are appropriate (eg, well-designed stopping rules in place when toxicity is an issue in clinical trials, identification of conflicts in statements about sample size and treatment allocation ratios). C-BSRF members also serve on the Data Safety Monitoring Committee (DSMC) to ensure that accrual, data collection, and compliance with monitoring rules are being followed. We successfully transformed the protocol review process in our CC: biostatistician’s approval now precedes approval by other units on investigator-initiated trials (IITs).

Pairing senior biostatisticians and cancer investigators (ie, teams) with common research interests promotes and facilitates collaboration. Biostatisticians gain enhanced understanding of cancer research themes (eg, immunology, genetics, epidemiology) and cancer investigators are introduced to, or enhance, their understanding of the role of biostatistics in their specific research area(s). This model of collaboration increases opportunities for mutual gain in publishing high-impact articles and obtaining multiple-PI grants. In our Core, for example, C-BSRF biostatisticians with expertise in the employment of multivariable prevalence ratios were key collaborators on a study designed to identify predictors of positive margins after definitive resection for gastric adenocarcinomas and adjuvant therapies, and biostatisticians with experience in comparative effectiveness methods designed a study investigating time-to-event outcomes in assessing the effects of postmastectomy radiation therapy by employing an immortal time bias methodology (13,14). A successful strategy for C-BSRF biostatisticians has been to review the research publications and grants of the CC’s investigators and, subsequently, to learn the emerging methodologies applicable to those fields of research.

## 4. Financial Support Plans

Successful C-BSRFs serve as a cost-effective resource for members of the CC by offering a balance of financial support plans to accommodate different types of projects and funding levels. Development and review of IIT protocols and grants should, ideally, be provided free of charge to investigators with support from CCSG. However, the expected arrangement is that when investigators using the subsidized resources of the Core write grants, they will provide the biostatisticians with whom they previously collaborated adequate FTE support on current research grant applications. The level of support budgeted should be clearly aligned with the scope of the work and account for the methodological implementation or development, data management, and statistical programming required for the project. We request that investigators budget at least 10% annually for statistical support on each RO1-level grant application.

Statistical support for services other than protocol and grant development and review can be arranged through a fee-for-service (charge-back) or long-term collaboration contract. Collaborating academic units can pay for statistical support on an hourly short-term basis (fee-for-service) or by funding a percentage of a biostatistician’s salary over the long term. Many CCs avoid fee-for-service systems, which can discourage use of the facility and inhibit creation of a collegial environment. Nonetheless, fee-for-service arrangements may be necessary and effective if there is high demand for biostatistical collaboration (15). Institutional support is essential for maintaining an effective, financially viable C-BSRF. A balance of funding through CCSG, intuitional support, and revenues through short- and long-term contract is most effective (7).

## 5. Statistical Support for a Majority of Grants at an Adequate Level of Support

Biostatistical support may not be necessary for some basic research grants, but it is essential for ensuring the scientific rigor of a majority of grant applications. We have found that while most investigators agree that biostatistical collaboration is needed, due to the demands on their time during the grant writing process, they often do not seek out the appropriate statistical collaborator. They frequently find the biostatistician so late in the process that the biostatistician does not have adequate time to understand the grant and write an appropriate statistical section. Often, an insufficient percentage of effort is budgeted for the biostatistician, and, as a result, the biostatistician does not collaborate at the optimal level.

We have employed three strategies for addressing this challenge. First, we have developed a Grant Support Policy for funding biostatisticians as co-investigators with an appropriate %FTE to alleviate disproportionate institutional support, and we have set guidelines that require receipt of all relevant materials and information at least four weeks prior to a submission deadline. While it is difficult to have to turn down our colleagues, we do say “no” when the guideline is not adhered to. With our response, we provide a letter of support describing our strengths and promising collaboration if the grant is funded and if the biostatistician can be added to the budget at that time. Second, we work directly with the grant administrators of each department to identify investigators without statistical support and to inform them of the Core’s free services, financial support models, and the Grant Support Policy. Third, we have worked effectively with our institution’s Grants and Contracts Office to build an electronic alert system that informs us whenever a grant with the keyword “cancer” is being submitted. Whenever we see that a biostatistician is not involved, we contact the PI directly to describe our services and emphasize the importance of inclusion of a biostatistician on the grant. We invite the investigator to engage in a conversation about their project and to share a copy of the grant. We often find that investigators understand the value of involving a biostatistician but do not have adequate budget for statistical support. In these situations, we engage the chair of the investigator’s department and/or the Director of the CC to make a case for matched funding for the biostatistician’s work through philanthropic resources or clinical revenue. We have saved a copy of this invitation letter on JNCI website.

## 6. Educational Activities for Biostatisticians

Successful C-BSRFs support CCs by providing education and training opportunities for the biostatisticians who will collaborate with CIs. Education and training for our C-BSRF biostatisticians includes workshops, journal clubs, online resources, and lab meetings and visits. The weekly Biostatistics Design Workshop consists of review of statistical designs and analysis of clinical trial protocols under consideration. The monthly Biostatistics Analysis Workshop discusses statistical methods and emphasizes adoption of novel methodologies: a biostatistician with a manuscript in progress presents the research and solicits feedback. The C-BSRF Director conducts a Biostatistics Grant Workshop, which engages TCI investigators in the development of ideas for data-heavy grant applications. C-BSRF members also attend workshops conducted by the ISMMS Faculty Development Office to enable them to understand what NIH, NCI, and National Science Foundation (NSF) reviewers are looking for in project design and analysis write-ups and to enable them to engage in informed discussions with their collaborators and trainees about grantsmanship.

Regular meetings of journal clubs for Core members and their collaborators promote understanding and adoption of new statistical methodologies. A pair of papers is presented, one reporting clinical or laboratory research and the other reporting on the statistical methodology employed, followed by discussion of novel methodologies to address potential limitations. Conference call options are available to enable biostatisticians and investigators working in different locations to participate.

We store online reference materials related to clinical trial protocol development on our C-BSRF server, which are readily available for training new biostatisticians and to ensure that all biostatisticians have a similar foundation in clinical trial research. These include guidelines for trial end point definitions, statistical designs for clinical trials, power calculations, boundary estimates, and operating characteristics of statistical stopping rules for toxicity and futility. Our biostatisticians also participate in laboratory tours and attend lab meetings to develop a deep understanding of the scientific questions and data sources that will inform their work with investigators. Often the biostatisticians are invited to share practices that improve the rigor and reproducibility of research.

## 7. Educational Activities for Investigators

Successful C-BSRFs also lead diverse educational activities for CIs to advance engagement, collaboration, and understanding of the role of biostatisticians in cancer research. Investigators are invited to attend all statistical workshops, as well as activities designed especially for them. Our educational and training activities for investigators include open houses, walk-in biostatistics clinics, mentoring and training, consulting, and online resources.

We sponsor a monthly lunchtime open house to bring investigators and biostatisticians together to explore new opportunities and plan how they may work together. Walk-in biostatistics clinics provide opportunities for in-depth discussions and more sustained collaborations. Dates, times, and locations are publicized on our website and through the CC’s ListServ. Mentoring and training young investigators is essential for the long-term success of CCs. Senior biostatisticians can serve as effective mentors for junior investigators on their NIH K Awards (Career Development Awards), providing guidance on the inclusion of novel statistical design and analytic methodologies to strengthen their applications. We also offer a lecture on clinical trial design for all incoming residents.

An important outcome of effective collaboration between biostatisticians and investigators is securing extramural grant funding. Biostatisticians must take an active role in communicating and advocating for the value of their work and unique contribution to the success of academic biomedical research (5). Statisticians can play a primary role, for example, in the design of studies based on the results of NIH RePORTER queries (16). NIH RePORTER includes data and analysis of NIH-supported research, which can help investigators consider areas where new grants could be submitted (https://projectreporter.nih.gov/reporter.cfm). Similarly, a biostatistician’s help with a search in pubmed.gov could identify gaps in the literature and acquisition of publicly available databases (https://healthcaredelivery.cancer.gov/seermedicare/; https://www.facs.org/quality-programs/cancer/ncdb).

Our Core also provides online resources and tools for training new collaborators on various aspects of conducting clinical research, including data management, tips on developing and writing protocols, conventional analytical methods and their implementation in common statistical software packages, guidelines on manuscript authorship, submission of data files to statisticians, and examples of common statistical problems to document and avoid. We consult with collaborations to link them with the right online resources to address their research needs.

## 8. Protected Time for Statisticians’ Career Growth

C-BSRF biostatisticians have dual responsibilities: supporting CIs and staying current with emerging statistical methods and research. Statistical expertise is becoming increasingly specialized, and C-BSRFs must maintain expertise in the fields crucial to the broad programmatic needs of CIs. A number of studies point to lack of expertise and education as a factor that hinders penetration of methodological advances (eg, Bayesian methods) in the design of confirmatory studies and exploitation of the potential advantages of adaptive clinical designs (17,18). We strongly advise a plan for providing protected time for biostatisticians’ career development to support learning about clinical and laboratory methods, development of proficiency in employing novel methodologies, participation in professional conferences, mentoring by senior biostatisticians, review of current literature, and pursuit of additional training (eg, webinars, continuing education courses) that may be of particular benefit for their collaborations with CIs (19). Protected time is essential for statisticians’ success in helping to achieve the CC’s goals, as well as advancing their own careers. Biostatisticians should track protected time and be able to show productivity that resulted from the time provided to them, such as papers on the development of novel methods or demonstrating deeper understanding of the data generation process of new technologies.

## 9. Development of an Informative Website

Transparency and standardization of every aspect of the C-BSRF’s work—guidelines and policies, activities, fees, and contracts—will inspire trust and promote collaboration. We recommend the development of a Core website linked to the CC. The website should post information about personnel, services, guidelines and policies, a calendar of activities, online scheduling process for appointments, and a schedule of open office hours (http://icahn.mssm.edu/research/institute-health-care-delivery/tci-biostatistics). The site should be referred to in correspondence between C-BSRF biostatisticians and CIs.

A key to productive working relationships between biostatisticians and CIs, as it is for most successful professional relationships, is clear communication (20,21). Guidelines posted on the site are intended to serve as a starting point for discussions about multiple aspects of the collaboration (eg, budgets, levels of collaboration, meeting planning, etc.), including appropriate attribution for co-authorship. We encourage early and explicit discussion of authorship in the research process. Our Core requires that biostatisticians be acknowledged as co-investigators if International Committee of Medical Journal Editors (ICMJE) criteria are met.

## 10. Optimizing Electronic Data Capture for Logging/Tracking Services

Electronic data capture (EDC) is required for NCI-funded CCs. We have found that development of a system with an expanded number of mandatory data input fields is most effective for accurately logging and tracking the Core’s services (https://erap.mssm.edu/Public/CancerRequestForServices.aspx). In addition to tracking work patterns and contribution to totality of effort, we capture the Investigator Satisfaction Survey through this system. We have saved the data items we collect on the JNCI website.

## Achieving Results

Implementation of these 10 strategies quickly enabled success for TCI’s C-BSRF from 2014 to 2017, with increases in service requests (131 to 200, 50% increase), funded grants (14 to 34, doubled), percentage of FTEs for biostatisticians in grants (1.2 FTE to 3.8 FTE, tripled) in addition to 1.6 FTEs on CCSG, and publications (11 to 47, quadrupled). 1) Full involvement of the Core’s Director in the CC’s leadership team increased engagement between biostatisticians and investigators, raised awareness of C-BSRF activities, and improved the quality and frequency of productivity reporting. Based on the Director’s discussions with CC leadership, biostatisticians were assigned to each of the Center’s eight “Disease Focus Groups,” and a more rigorous process for statistical review of IITs was developed (median weeks to approval has decreased from two to one since inception). 2) Ensuring that biostatisticians were fully dedicated to cancer resulted in improved quality of research by promoting highly focused collaborative efforts, including publication of methodology papers on novel ways to design, analyze, and meta-analyze (22–27). 3) Efforts to establish programmatic collaborations included expansion of the Core’s staff and recruitment of new personnel, providing greater breadth and depth to support CI projects (28–31), and identifying Investigator-Statistician-Decision Scientist teams to boost the production of high-impact publications (32–34). 4) Implementing options for cost-effective financial support and developing a grant support policy for funding biostatisticians increased the percentage of FTE on grants, significantly offsetting disproportionate institutional support. 5) Efforts to expand statistical support on grants increased the number of submitted grants and increased the percentage of FTE for biostatisticians. Approximately 57% of CIs respond to our letter of introduction offering statistical support for current grant applications, and many who do not respond approach us for support on future grant submissions. 6) Proper educational training activities and mentoring of biostatisticians improved the quality of clinical research and efficiency of the protocol review process. 7) Offering educational activities for investigators resulted in improved communication between biostatisticians and young CIs. 8) Providing protected time for C-BSRF biostatisticians enabled career development, including training in new statistical methodologies and submission of grants for development of novel statistical methods to NIH and Patient-Centered Outcomes Research Institute (PCORI). 9) Development of an informative website ensured transparency and standardization in all aspects of the Core’s work, facilitating communication and engagement in collaborative efforts. 10) Development and steady use of an EDC system enhanced logging and tracking of collaborative projects, provided greater transparency and accuracy of the mandatory data reporting required by NCI for CC designation, and provided recognition of the quantity and variety of work performed by the C-BSRF team. Feedback from satisfaction surveys has been positive: “incredibly thoughtful and responsive,” “added tremendous amount to the proposal,” and “learned a lot and felt that the input from the biostatisticians was valuable,” with the predominant negative comment involving issues with longer-than-expected wait times for project completion, an issue, that will be resolved by further expansion of C-BSRF.

We hope the essential elements described in this commentary will be valuable for new and reforming C-BSRFs. They can be adapted and adjusted for CCs of various sizes and with different types of cancer research programs. Our website survey saved as supplemental materials on JNCI website, provides centralized access to all C-BSRFs, a type of resource that has been shown to be beneficial for Clinical Translational Science Centers (35). The survey can help new or reforming C-BSRFs identify a CC similar to their own as they begin to build their resources. We recommend direct communication with the Directors of C-BSRFs with CCs similar to their own to learn more about the strategies that have made their cores successful.

## Notes

Affiliations of authors: Institute for Healthcare Delivery Science, Mount Sinai Health System, New York, NY (MM); Center for Biostatistics, Department of Population Health Science and Policy (MM, ELM, OU), Department of Oncological Sciences (RP), Medicine, Hematology and Medical Oncology (RP), and Biostatistics Shared Resource Facility (MM, ELM, OU), Tisch Cancer Institute (RP), Icahn School of Medicine at Mount Sinai, New York, NY.

We wish to acknowledge all of the members of the TCI-BSRF: Parul Agarwal, PhD, Kezhen Fei, MS, Bart Ferket, MD, PhD, Liangyuan Hu, PhD, Rachel Jia, PhD, Jung-Yi (Joyce) Lin, MS, John Mandeli, PhD, Meng Ru, MS, Xiaoyu, Song, PhD, Gary Winkel, PhD, Rowena Yip, MS, Hsin-Hui (Vivien) Huang, MS, PhD, MD, Nicole Zubizarreta, MPh.

Personnel involved in the development of TCI-BSRF who have since left ISSMS or are not part of the Core: James Godbold, PhD (served as Associate Director), Jashvant Poeran, MD, PhD, Ksenia Gorbenko, PhD, Catherine Craven, PhD, Ashley Olson, MA, Katherine Kirkwood, MSc.

We also acknowledge members of our external advisory board, with special thanks to the chair, Dr. Edward Benz (President, Dana-Farber/Harvard Cancer Center) and Dr. Yu Shyr (Chair, Department of Biostatistics, Vanderbilt University School of Medicine; Director, Quantitative Science Shared Resource, Vanderbilt-Ingram Cancer Center) for providing key guidance for reform and strong support for increased resource allocation to the BSRF.

The authors wish to acknowledge the support of the NCI CCSG -30 CA10652-01, ISSMS, and its past principal investigator, Steven J. Burakoff, MD. Dr. Burakoff was the Founding Director of TCI. He currently serves as the Dean of Cancer Innovation, ISMMS and is Professor of Oncological Sciences, Medicine, Hematology and Medical Oncology. Dr. Burakoff is also Chief of Pediatric Oncology.

We also thank Ms. Sima Rabinowitz for expert editorial assistance.

Dr. Madhu Mazumdar acknowledges Dr. Colin Begg (Chairman, Department of Epidemiology and Biostatistics at Memorial Sloan Kettering Cancer Center) for providing excellent supervision and training on biostatistical collaboration and resource administration during her first post-doctoral position at MSKCC in 1991. The C-BSRF model and strategies proposed here are closely based on those established by Dr. Begg at MSKCC.

## Supplementary Material

Supplementary Data
