# Supplementary material for: Ten Essential Practices for Developing or Reforming a Biostatistics Core for a NCI Designated Cancer Center
Source: JNCI Cancer Spectr. 2018 Apr 28;2(1):pky010. doi: 10.1093/jncics/pky010 (PMC6649702; doi:10.1093/jncics/pky010)
Supplement: Supplementary Data [file pky010_supp.pdf]

Supplemental Materials:

Ten Essential Practices for Developing or Reforming a Biostatistics Core for a NCI Designated Cancer Center

Madhu Mazumdar, Erin L. Moshier, Umut Ozbek, Ramon Parsons

		Survey of NCI Cancer Centers	Acronyms: CC:Cancer Center; CP: Comprehensive CC; CL: Clinical CC; L: Laboratory CC	
ID	Status	Name of cancer center	URL of Cancer Center website	Biostat Core URL
1	CP	Dana Farber/Harvard CC	http://www.dfhcc.harvard.edu/	http://www.dfhcc.harvard.edu/research/core-facilities/biostatistics/
2	CL	Koch Institute-MIT	https://ki.mit.edu/	https://ki.mit.edu/sbc/bioinformatics/services
3	CL	Jackson Lab. CC	https://www.jax.org/research-and-faculty/tools/the-jackson-laboratory-cancer-center	https://www.jax.org/research-and-faculty/tools/scientific-research-services/computational-sciences/biostatistics-and-statistical-genetics
4	CP	Dartmouth-Norris Cotton CC	http://cancer.dartmouth.edu/index.html	http://cancer.dartmouth.edu/researchers/biostatistics-resources.html
5	CP	Yale cancer Center	http://www.yalecancercenter.org/	http://www.yalecancercenter.org/research/resources/BSRservices.aspx
6	CP	U Penn-Abramson Cancer Center	https://www.pennmedicine.org/cancer	https://www.pennmedicine.org/cancer/cancer-research/for-researchers/shared-resources/biostatistics-core
7	CP	Roswell Park Cancer Center	https://www.roswellpark.org/	https://www.roswellpark.edu/shared-resources/biostatistics
8	CL	Albert Einstein Cancer Center	http://www.einstein.yu.edu/centers/cancer/	http://www.einstein.yu.edu/research/shared-facilities/cores/44/biostatistics/
9	CP	Fox Chase Cancer Center	https://www.foxchase.org/	https://www.foxchase.org/research-education/services-support/computing-and-information/biostatistics-and-bioinformatics
10	CP	Herbert Irving Cancer Center (Includes Columbia Med. Center and NY Pres)	http://cancer.columbia.edu/	http://cancer.columbia.edu/research-group/biostatistics
11	CL	Tisch Canct Inst-Mount Sinai	http://icahn.mssm.edu/research/tisch	http://icahn.mssm.edu/research/tisch/resources/biostatistics
12	CP	NYU Langone-Laura and Isaac Perlmutter CC	https://nyulangone.org/locations/perlmutter-cancer-center	http://www.med.nyu.edu/pophealth/divisions/biostatistics
13	CP	Memorial Sloan Kettering Cancer Center	https://www.mskcc.org/	https://www.mskcc.org/departments/epidemiology-biostatistics/biostatistics
14	CP	Rutgers Cancer Institute of New Jersey	http://www.cinj.org/	https://www.cinj.org/research/biometrics
15	CP	Johns Hopkins Medicine-Sidney Kimmel Comprehensive Cancer Center	http://www.hopkinsmedicine.org/kimmel_cancer_center/	http://www.hopkinsmedicine.org/kimmel_cancer_center/research_clinical_trials/research/shared_resources/biostatistics_core.html
16	CL	Thomas Jefferson University-Sidney	http://www.kimmelcancercenter.org/cancer-center.html	http://www.kimmelcancercenter.org/cancer-center/research/shared-resources/biostatistics.html
17	CP	University of Pittsburgh Hillman CI	http://upci.upmc.edu/	http://upci.upmc.edu/bf/
18	L	Wistar Institute CC	https://wistar.org/research-discoveries/wistar-institute-cancer-center	https://www.publichealth.pitt.edu/biostatistics
19	CP	Wayne State-Detroit-Barbara Ann Karmanos CC	https://www.karmanos.org/about/wayne-state-university-partnership	http://www.karmanos.org/Biostats
20	CP	Case Western-Case Comprehensive CC	http://cancer.case.edu/	http://cancer.case.edu/research/sharedresources/biostatistics/
21	CP	The Ohio State University Comprehensive Cancer Center	https://cancer.osu.edu/	https://cancer.osu.edu/research-and-education/shared-resources/biostatistics
22	CL	Indiana University Melvin and Bren Simon Cancer Center	http://www.cancer.iu.edu/	http://www.cancer.iu.edu/research-trials/facilities/biostatistics/index.shtml
23	CL	Purdue University Center for Cancer Research	https://www.cancerresearch.purdue.edu/	Joint with Indiana Univ Cancer center https://apps.cancer.iu.edu/bioinformatics/
24	CP	Northwestern-Robert H. Lurie CCC	http://cancer.northwestern.edu/	http://cancer.northwestern.edu/research/shared_resources/quantitative_data_sciences/index.cfm
25	CP	The Univ. of Chicago Medicine CCC	https://cancer.uchicago.edu/	http://health.bsd.uchicago.edu/Research/BiostatisticsLaboratory
26	CP	Umich-Comprehensive Cancer Center Michigan Medicine	https://www.mcancer.org/	http://www.mcancer.org/research/shared-resources-and-cores/biostatistics
27	CP	Univ. Of Wisconsin Carbone Cancer Center	https://www.uwhealth.org/uw-carbone-cancer-center/cancer/10252	https://cancer.wisc.edu/research/resources/bsr/
28	CL	Wash U-Alvin J. Siteman Cancer Center	https://siteman.wustl.edu/	https://siteman.wustl.edu/research/shared-resources-cores/biostats-core/
29	CL	U Nebraska-Fred and Pamela Buffet CC	https://www.unmc.edu/cancercenter/	https://www.unmc.edu/cancercenter/research/index.html
30	CP	U Iowa-Holden CCC	https://uihc.org/primary-and-specialty-care/holden-comprehensive-cancer-center	https://uihc.org/biostatistics-core
31	CP	U Minnesota-Masonic Cancer Center	https://www.cancer.umn.edu/	https://www.cancer.umn.edu/for-researchers/shared-resources/biostatistics-bioinformatics
32	CP	Mayo Clinic	https://www.mayoclinic.org/departments-centers/mayo-clinic-cancer-center	http://www.mayo.edu/research/centers-programs/cancer-research/shared-resources-core-facilities-services/biostatistics
33	CL	Univ. Kansas CC	https://www.kucancercenter.org/	http://www.kucancercenter.org/cancer-research-and-education/shared-resources/biostatistics-informatics
34	CP	Duke CI	http://www.dukecancerinstitute.org/	http://www.dukecancerinstitute.org/biostatistics
35	CP	Georgetown Lombardi CCC	https://lombardi.georgetown.edu/#	https://lombardi.georgetown.edu/research/sharedresources/bbsr
36	CL	U South Carolina-Hollings CC	http://www.hollingscancercenter.org/index.html	http://www.hollingscancercenter.org/research/shared-resources/biostatistics/index.html
37	CL	Virginia Commonwealth U-Massey CC	https://www.massey.vcu.edu/	https://www.massey.vcu.edu/research/cores/biostats/
38	CP	Moffitt CC	https://moffitt.org/	https://moffitt.org/research-science/shared-resources/biostatistics/
39	CP	UNC Lineberger CCC	https://unclineberger.org/	http://unclineberger.org/research/core-facilities/research/core-facilities/biostats/overview
40	CP	Wake Forest CCC	http://www.wakehealth.edu/Comprehensive-Cancer-Center/	http://www.wakehealth.edu/Research/Comprehensive-Cancer-Center/BB/Biostatistics-and-Bioinformatics.htm
41	CP	Emory-Winship CI	https://winshipcancer.emory.edu/index.html	https://winshipcancer.emory.edu/research/shared-resources/biostatistics-bioinformatics.html
42	CL	U Kentucky-Markey CC	https://ukhealthcare.uky.edu/markey-cancer-center	https://ukhealthcare.uky.edu/markey-cancer-center/research/bbsrf
43	CP	St. Judes Children Research	https://www.stjude.org/	https://www.stjude.org/research/shared-resources/biostatistics-shared-resource.html
44	CP	UAB CCC	http://cancercenter.uab.edu/	http://www3.ccc.uab.edu/research/shared-facilities/biostatistics-and-bioinformatics/
45	CP	Vanderbilt-Ingram CC	https://www.vanderbilthealth.com/cancer/34703	http://www.vicc.org/biostatistics/
46	CL	UT San Antonio CC Research Ctr	https://makelivesbetter.uthscsa.edu/cancercenter	http://www.uthscsa.edu/patient-care/ctrc/biostatistics
47	CP	Baylor U-Dan L Duncan CCC	https://www.bcm.edu/centers/cancer-center	https://www.bcm.edu/centers/cancer-center/research/shared-resources/biostatistics-and-informatics
48	CP	UT Southwestern TX-Harold Simmons	http://www.utsouthwestern.edu/simmons/	http://www.utsouthwestern.edu/simmons/shared-resources/biostatistics.html
49	CP	UT MD Anderson	https://www.mdanderson.org/	https://www.mdanderson.org/research/departments-labs-institutes/departments-divisions/biostatistics/research.html
50	CP	UC Irvine Chao Family CCC	http://www.cancer.uci.edu/	https://www.cancer.uci.edu/biostatistics/index.asp
51	CP	Fred Hutch of U. of Washington	http://www.cancerconsortium.org/en/about.html	http://sharedresources.fredhutch.org/core-facilities/biostatistics-resource
52	CL	Oregon Health Science U-Knight Cancer Institute	http://www.ohsu.edu/health/cancer/index.html	http://www.ohsu.edu/xd/health/services/cancer/research-training/shared-resources/biostatistics.cfm
53	CP	UC Davis Cancer Center	http://www.ucdmc.ucdavis.edu/cancer/	http://www.ucdmc.ucdavis.edu/cancer/research/sharedresources/biostatistics.html

Items Included in Electronic Data Capture

- Disease Focus Group (DFG)
- Service Requested
  - Data management
  - Database Construction
  - Grant Development
  - Grant Review
  - Networking with Researchers
  - New Course Development
  - Presentation of Research Seminar
  - Programming Tool Development
  - Protocol Development
  - Protocol Review
  - Statistical Analysis Plan for a Grant Proposal
  - Statistical Data Analysis
  - Statistical Methods Development
  - Study Design
  - Teaching (lecture, journal club, workshop)
  - First
- First/Last Name of Requester
- Requestor Email
- Requestor Department
- Cancer Center membership status of Requester
  - I am a member
  - My department chair/lab PI is a member
  - My collaborator is a member
- Name of Cancer Center member (select name from supplied list of names on drop-down menu)
- Title of Requestor
  - Professor
  - Associate Professor
  - Assistant Professor
  - Adjunct Professor
  - Fellow
  - Resident
  - Student
- Affiliate (select name from supplied list of affiliate institutions on drop-down menu)
- Expected Outcome
  - Abstract
  - Book Chapter
  - Grant
  - Manuscript
  - Protocol
  - Other
- Have you or any of your team members received a peer reviewed grant in the last three years (Yes/No)
- Is this project supported by a peer-reviewed grant (Yes/No)
- Is this request a revisit to the CC-BSRF? (Yes/No)
- Requestor Comment (free handed text provided by requestor about nature of request)

Example of Email Describing the Benefit of Increasing Biostatisicians' Involvement in Grant Writing and Submission with Adequate FTE

Dear '*Investigator Name inserted here*',

Hope you are well.

We received notification from GCO that you are submitting a grant titled '*Grant Title Inserted Here*' to '*Sponsor Name Inserted Here'*. Would it be possible for you to provide us with a brief description of your grant aims (or the full grant if that is easier) so we can determine together whether your grant application would benefit from biostatistical support?

It is well documented that biostatistical collaboration increases chances of funding[1-5] and success with publication[6,7]. Therefore, using resources from our NCI designated cancer center, an expansive and efficient biostatistics shared resource facility is recommended. Training and recruitment of biostatisticians is being targeted to cover all fields of cancer research performed by investigators at '*the institution'*. If your grant focuses on a field of research that extends beyond our expertise, we want to know so we can direct our recruitment and training efforts appropriately. Our goal is to ensure our investigators are adequately supported for their biostatistical needs and biostatisticians are adequately budgeted on the grants.

Sincerely,
xxx, PhD
Director, Biostatistics Shared Research Facility, xxx Cancer Institute
Professor, Biostatistics, Dept. of xxx

[1] Zhang G, Chen JJ. Biostatistics Faculty and NIH Awards at U.S. Medical Schools. The American statistician. Feb 2015;69(1):34-40.
[2] Berg KM, Gill TM, Brown AF, Zerzan J, Elmore JG, Wilson IB. Demystifying the NIH Grant Application Process. Journal of general internal medicine. 2007;22(11):1587-1595.
[3] Inouye SK, Fiellin DA. An evidence-based guide to writing grant proposals for clinical research. Annals of internal medicine. Feb 15 2005;142(4):274-282.
[4] Bordage G, Dawson B. Experimental study design and grant writing in eight steps and 28 questions. Medical education. Apr 2003;37(4):376-385.
[5] Wang L, Byrne D, Nian H, Yu C, Harrell F. Importance of Biostatistics in Academic Research Success. Paper presented at: Joint Statistical Meeting, https://www.amstat.org/meetings/jsm/2015/onlineprogram/AbstractDetails.cfm?abstractid=3174472015;<http://www.amstat.org/meetings/jsm/2015/onlineprogram/AbstractDetails.cfm?abstractid=3174472015;> Seattle, WA.
[6] Mazumdar M, Banerjee S, Van Epps HL. Improved reporting of statistical design and analysis: guidelines, education, and editorial policies. Methods in molecular biology. 2010;620:563-598.
[7] Bordage G. Reasons reviewers reject and accept manuscripts: the strengths and weaknesses in medical education reports. Academic medicine : journal of the Association of American Medical Colleges. Sep 2001;76(9):889-896.
